# Machine learning-based analysis of overall stability constants of metal–ligand complexes

**DOI:** 10.1038/s41598-022-15300-9

**Published:** 2022-07-25

**Authors:** Kaito Kanahashi, Makoto Urushihara, Kenji Yamaguchi

**Affiliations:** 1 Innovation Center, Mitsubishi Materials Corporation, 1002-14 Mukohyama, Naka, Ibaraki 311-0102 Japan; 2 Present Address: Department of Applied Physics, Nagoya University, Furo-cho, Chikusa-ku, Nagoya, 464-8603 Japan

**Keywords:** Cheminformatics, Organic molecules in materials science

## Abstract

The stability constants of metal(M)-ligand(L) complexes are industrially important because they affect the quality of the plating film and the efficiency of metal separation. Thus, it is desirable to develop an effective screening method for promising ligands. Although there have been several machine-learning approaches for predicting stability constants, most of them focus only on the first overall stability constant of M-L complexes, and the variety of cations is also limited to less than 20. In this study, two Gaussian process regression models are developed to predict the first overall stability constant and the *n*-th (*n* > 1) overall stability constants. Furthermore, the feature relevance is quantitatively evaluated via sensitivity analysis. As a result, the electronegativities of both metal and ligand are found to be the most important factor for predicting the first overall stability constant. Interestingly, the predicted value of the first overall stability constant shows the highest correlation with the *n*-th overall stability constant of the corresponding M-L pair. Finally, the number of features is optimized using validation data where the ligands are not included in the training data, which indicates high generalizability. This study provides valuable insights and may help accelerate molecular screening and design for various applications.

## Introduction

Metal(M)-ligand(L) complexes are one of the most important compounds in modern industry, such as electro-/electroless plating^1^, selective separation of rare or toxic elements^2,3^, drug design^4^, and analytical chemistry^5^. Among various properties of M-L complexes, their stability constants in an aqueous solution, which imply the binding strength between M and L, play an essential role in those industrial fields. For example, since the stability constants determine the concentration of free metal cations in the solution, they affect the quality of the plating film and the process efficiency of separating target metals. In the solution with a mixture of M and L, M-L_n_ complexes are formed through step-by-step ligand addition to the metal cation as follows:

1 $$ \begin{array}{*{20}c}    {M + L \leftrightarrow M - L \Rightarrow K_{1}  = \frac{{\left[ {{\text{M - L}}} \right]}}{{\left[ {\text{M}} \right]\left[ {\text{L}} \right]}},}  \\   \end{array}  $$

2 $$ \begin{array}{*{20}c}    {M - L_{{n - 1}}  + L \leftrightarrow M - L_{n}  \Rightarrow K_{n}  = \frac{{\left[ {{\text{M  -  L}}_{{\text{n}}} } \right]}}{{\left[ {{\text{M  -  L}}_{{{\text{n - 1}}}} } \right]\left[ {\text{L}} \right]}},}  \\   \end{array}  $$

where *K*_*n*_ corresponds to the equilibrium constant. Using Eqs. (1) and (2), the *n*-th overall stability constant *β*_*n*_ is defined as:

3 $$ \begin{array}{*{20}c} {\beta_{n} = \log K_{1} \times \cdots \times K_{n} = \log \frac{{\left[ {{\text{M - L}}_{n} } \right]}}{{\left[ {\text{M}} \right]\left[ {\text{L}} \right]^{n} }} .} \\ \end{array} $$

Furthermore, *β*_*n*_ intrinsically depends not only on the constituent elements of the ligand but also on its molecular structure. Considering an enormous number of M-L combinations in the chemical space, it is impractical to perform measurements of the overall stability constants for all candidates to find promising ligands. Therefore, there has been a great need for efficient methods predicting stability constants of arbitrary M-L pairs to accelerate either the design or screening of ligands for specific metals.

Over the past decades, machine learning approaches have been employed to predict various properties of M-L complexes, such as the spin-state splitting^6^ and the volcano plot^7^. In general, there are two ways of predicting the properties of M-L complexes by machine-learning techniques: using the features calculated from the M-L complex itself, which are usually derived from the first principles calculation, or using the features calculated from M and L. Because it is not obvious what three-dimensional molecular structure the M-L complex will form in an aqueous solution, most of the machine-learning studies aiming to predict overall stability constants were developed by compositional and/or topological features of metals and ligands^8–18^. Here, details of previous works, which are also issues to be resolved in this study, are summarized. First, the variety of cations needs to be expanded because most of the previous reports covered a limited set of less than 20 metals. Second, a machine-learning model that predicts multi-order *β*_*n*_ needs to be developed because previous studies focused mainly on *β*_1_. Third, the regression models in the previous works cannot conduct Bayesian optimization, which is a powerful technique to find the optimum candidate^19,20^. Since the Bayesian optimization requires both the predicted value and predicted variance to choose the promising condition, Gaussian process regression (GPR) is the most suitable. GPR is one of the nonlinear and nonparametric regression algorithms and has been used to derive not only material and molecular properties but also force fields for molecular dynamics simulation^19^. To date, there is no report on developing GPR models for predicting stability constants. Forth, the interpretability of the machine-learning model needs to be improved. If we evaluate the relevance of both cation and ligand properties on overall stability constants, the results can be compared with physical understanding. Although Chaube et al. reported the feature importance of both cations and ligands through the analysis of their machine-learning models, such as random forest feature importance and permutation importance, none of the cation features were even in the top 10, despite *β*_*n*_ being determined by the interaction between cation and ligand^8^. Moreover, to our knowledge, it remains unclear what kinds of properties are critical for multi-order *β*_*n*_. Thus, quantitatively predicting the overall stability constants of arbitrary M-L pairs in the diverse chemical space remains a challenge.

In this work, we overcome the above four obstacles. We collected experimental results for overall stability constants from existing publications to prepare an extremely large training dataset containing 19,810 data points. This original dataset is composed of two sub-datasets: one has 13,559 data points for *β*_1_ of 57 cations and the other one has 6251 data points for multi-order *β*_*n*_ (*n* = 2–6) of 50 cations. Using compositional and topological features of both cations and ligands as the descriptor, we trained a GPR model for predicting *β*_1_. Subsequently, we developed another GPR model for predicting multi-order *β*_*n*_ by employing the predicted *β*_1_ values of the corresponding M-L pairs as one of the features. To improve the interpretability of our models, we performed a sensitivity analysis. Consequently, it was found that electrical features, such as electronegativity and ionic properties, of both cations and ligands are the most important for predicting *β*_1_. Furthermore, the predicted *β*_1_ value was found to have the strongest relevance to predicting multi-order *β*_*n*_ of the corresponding M-L pair. Note that these results are consistent with the physical understanding of the complex formation. Finally, the GPR models exhibited high generalizability for ligands for which data were not contained in the training datasets and those located near the edge of the applicability domain. Our machine-learning modeling and analysis provide novel insights for complex formation and are expected to provide a pathway to accelerating molecular design and screening for various applications.

## Results

### Visualization of the initial dataset

**Figure 1:**
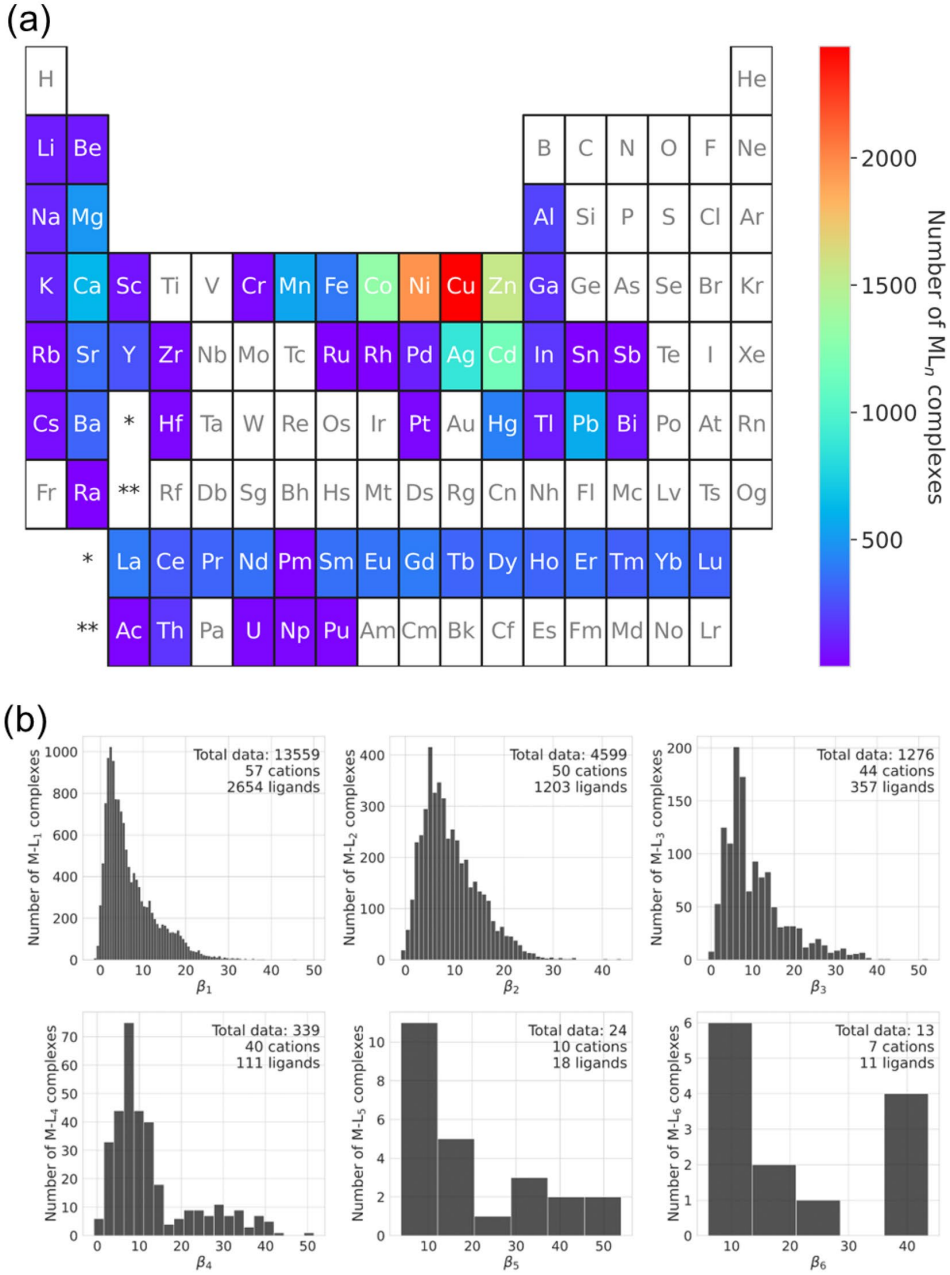
(**a**) Total experimental results of each cation in the initial dataset, which is composed of 57 cations and 2706 ligands. (**b**) Distribution of each *β*_*n*_ in the initial dataset. The total amount of data, cations, and ligands are also displayed.

Details on how the initial dataset was prepared are described in the Methods section. As one of the descriptions for the chemical space, Fig. S1 shows the distribution of the molecular weights of the ligands. To our knowledge, there is no previous study on the prediction of overall stability constants using such a large dataset (19,810 data points containing 57 cations). Due to the increased number of data points and cation species, the generalizability of the machine-learning model is expected to improve. Figure 1a summarizes the total number of entries for each cation. Note that our dataset encompasses diverse metals, including alkali metals, alkaline-earth metals, noble metals, transition metals, and rare-earth metals. One can see that there is a large amount of data for Cu^2+^, Ni^2+^, Zn^2+^, Co^2+^, Cd^2+^, Ag^+^, and Ca^2+^, accounting for 50% of the total data. Figure 1b shows the distribution and total numbers of data, cations, and ligands for each *β*_*n*_. As shown in Fig. 1b, although there are a lot of experimental results up to *β*_4_, the amount for *β*_5_ and *β*_6_ is quite small. In this study, due to this limitation on the data for *β*_5_ and *β*_6_, we created two machine-learning models: a model for predicting the first overall stability constant *β*_1_ and a model for predicting multi-order *β*_*n*_ (*n* = 2–6) using appropriate descriptors (see Methods section).

### Sensitivity analysis and optimization of the GPR model for predicting ***β***_1_

**Figure 2:**
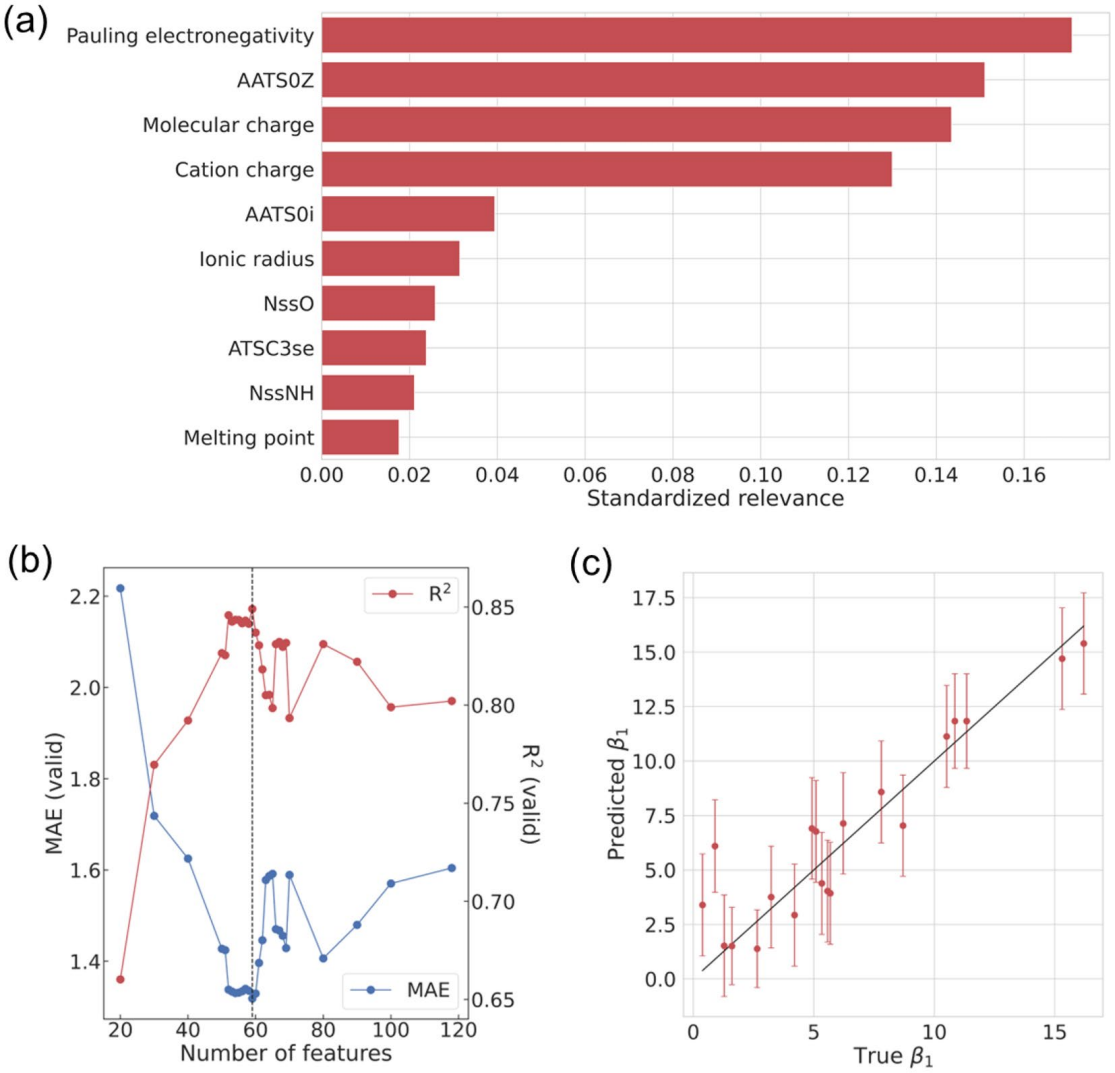
(**a**) The top 10 highest ranked features through sensitivity analysis using a Kullback–Leibler divergence as a measure for predicting *β*_1_. (**b**) Predictive performance for the validation samples as a function of the number of features. Features are arranged in descending order of relevance. The black dashed line corresponds to the top 59 features. (**c**) Parity plot between true and predicted *β*_1_ values of the validation data using the best GPR model. Error bars indicate 1σ uncertainty of the predicted value.

We prepared a total of 118 features to create a GPR model for predicting *β*_1_ in this study (see the Methods section). Feature selection is critically important for creating a machine-learning model with high predictive performance. In GPR, although the relevance of each feature is usually interpreted as the inverse of its length scale parameter, some previous reports have pointed out that this approach sometimes does not work well^21–23^. Accordingly, we evaluated the relevance of each feature via sensitivity analysis using a Kullback–Leibler (KL) divergence as a measure^23^. We set the perturbation to 0.001 during calculation. Figure 2a shows the standardized relevance of the 10 highest-ranked features using the GPR model with optimized hyperparameters that uses full feature *β*_1_ (all results are listed in Supplementary Information S2). The total contribution of these 10 features reaches 0.755. As shown in Fig. 2a, the Pauling electronegativity of metals is the most relevant feature for predicting *β*_1_. Moreover, ionic properties, such as molecular charge, cation charge, and ionic radius, are also highly relevant. Among the ligand features, Moreau–Broto autocorrelation of topological structure features (AATS0Z, AATS0i, and ATSC3se) and fragmental features (NssO and NssNH) are in the top 10 features. AATS0Z, AATS0i, and ATSC3se are computed based on a molecular graph and depend on atomic number, ionization potential, and the Sanderson electronegativity of the elements in the ligand, respectively. NssO and NssNH correspond to the number of chemical structures, such as -O- and -NH-, respectively. In particular, oxygen and nitrogen become coordination sites due to their high electronegativity, suggesting that the relevance scores of NssO and NssNH are high.

Next, we performed feature optimization of the *β*_1_ GPR model while monitoring the predictive performance. Note that usual cross-validation techniques do not reproduce the original purpose of predicting unknown ligands because it is unavoidable for common ligands to remain in both training and validation data, which may result in an overestimation of the predictive performance. Thus, we extracted 20 appropriate ligands based on the applicability domain of our model and calculated mean absolute error (MAE) and coefficient of determination (R^2^) for them. The selection rule for the validation samples is described in Supplementary Information S3, and we would like to emphasize that the 20 selected ligands are not contained in the training dataset. Figure 2b summarizes the predictive performance for the validation data using the GPR model as a function of the descriptor dimension. The features were arranged in descending order of relevance scores, as shown in Fig. 2a. Consequently, it is concluded that the best features for predicting *β*_1_ are the top 59 features (MAE: 1.31, R^2^: 0.84), which are composed of 8 cation features, 49 ligand features, and 2 experimental conditions. Furthermore, Fig. 2c shows the parity plot between true and predicted *β*_1_ values of the validation data using the best GPR model, implying the high generalizability of our model. The cross-validations of the feature-optimized GPR model for predicting *β*_1_ also indicated good predictive performance (see Supplementary Information S5).

### Sensitivity analysis and optimization of GPR model for predicting multi-order ***β***_n_

**Figure 3:**
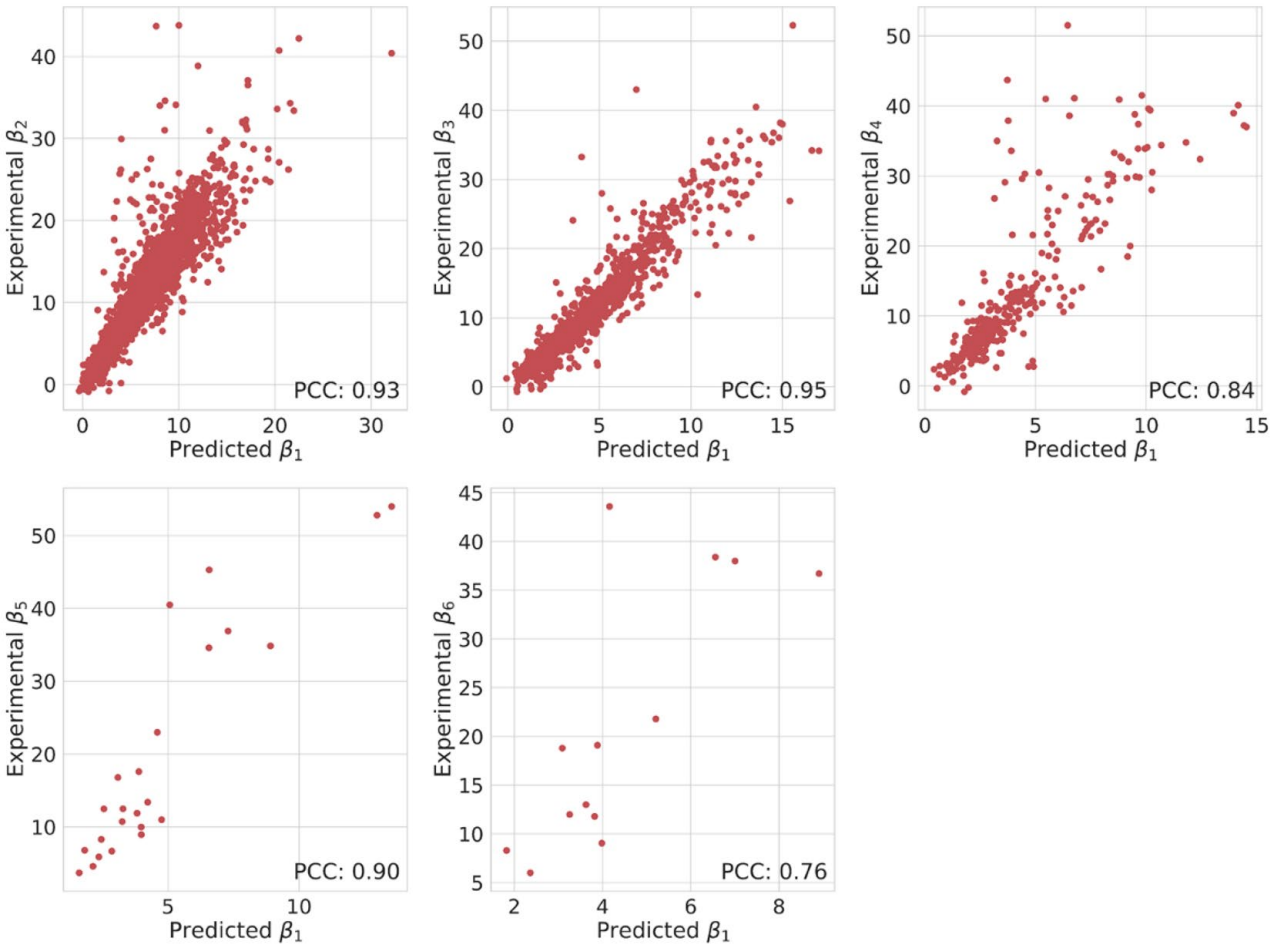
Relationships between experimental multi-order *β*_*n*_ and predicted *β*_1_ of the corresponding M-L pair. The results of Pearson correlation coefficients (PCC) are also displayed.

As demonstrated in the prediction of *β*_1_, the feature selection is critical in predicting multi-order overall stability constants *β*_*n*_ as well. For Co^2+^, Ni^2+^, and Cu^2+^ in particular, it has been reported that there are linear correlations between *β*_1_ and *β*_2_^16^. In the present study, we demonstrate that the strong correlations between *β*_1_ and *β*_*n*_ are observed not only in other cations but also in higher coordination numbers. Figure 3 summarizes the relationship between experimental multi-order overall stability constants *β*_*n*_ and predicted *β*_1_ values of the corresponding M-L pair. Note that not all M-L pairs for *β*_*n*_ are contained in the dataset for *β*_1_. As shown in Fig. 3, one can see a strong correlation between each of the true *β*_*n*_ and predicted *β*_1_ values, resulting in large positive Pearson correlation coefficients (PCC). Therefore, the predicted *β*_1_ for the M-L_n_ complex is expected to be a significantly effective feature for predicting multi-order *β*_*n*_. Because we have succeeded in predicting *β*_1_ by combining features of cations and ligands, it is thought to be feasible to predict multi-order *β*_*n*_ by using features of M-L complex and L. Consequently, we prepared a total of 60 features to create a GPR model for predicting multi-order *β*_n_ in this study (see the Methods section).

**Figure 4:**
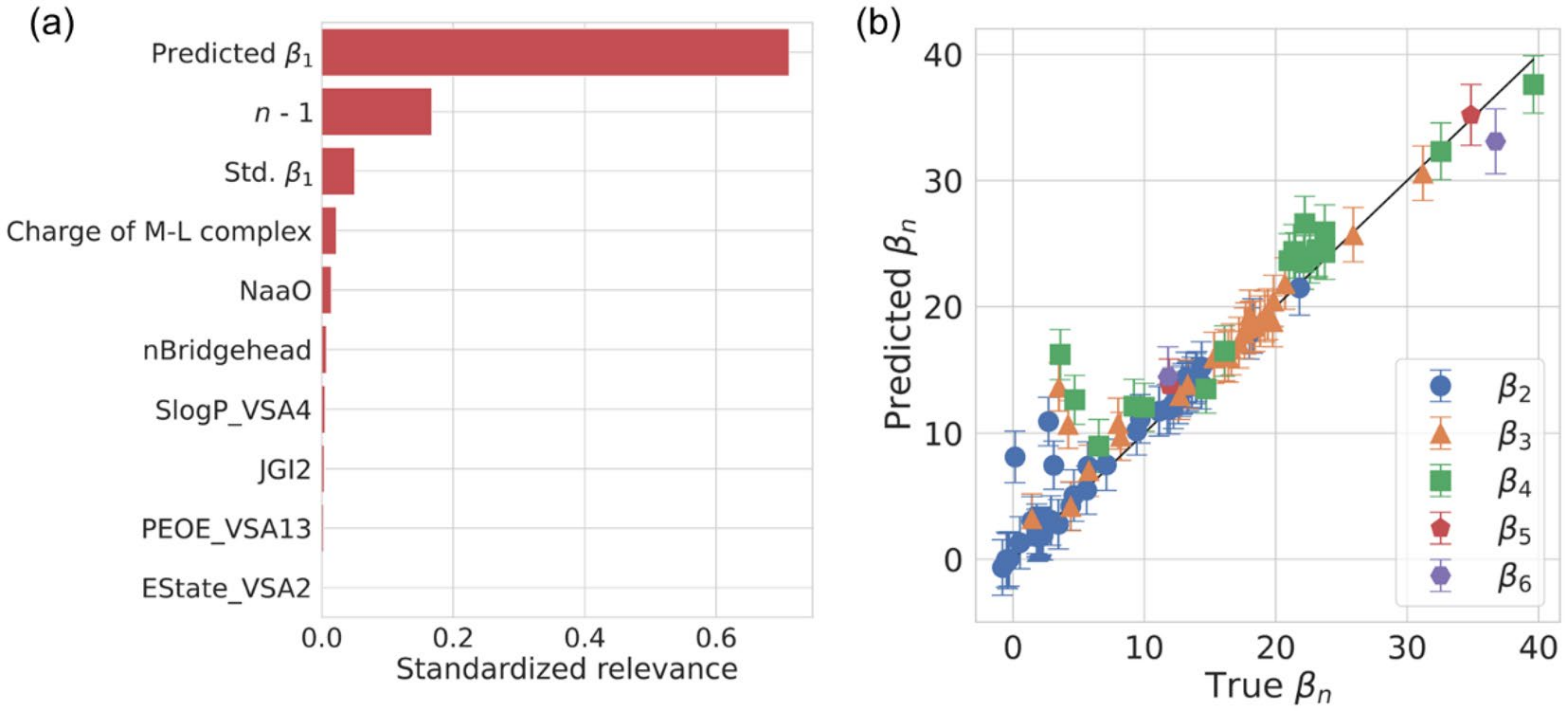
(**a**) The top 10 highest ranked features through sensitivity analysis using a Kullback–Leibler divergence as a measure for predicting multi-order *β*_*n*_. (**b**) Parity plot between true and predicted multi-order *β*_*n*_ values of the validation data using the best GPR model. Error bars indicate 1σ uncertainty of the predicted value.

Similar to the *β*_1_ GPR model, Fig. 4a shows the standardized relevance of the top 10 highest-ranked features using the full-feature-used *β*_n_ GPR model with optimized hyperparameters (the full result is provided in Supplementary Information S4). The total contribution of these features reaches 0.986. As shown in Fig. 4a, it is obvious that the predicted *β*_1_ for the M-L pair is the most important feature. NaaO and nBridgehead are fragmental features, which are defined as the number of chemical structures like –O– among aromatic rings and the number of bridgehead atoms, respectively. The *X*_VSA*Y*, such as SlogP_VSA4, PEOE_VSA13, and EState_VSA2, is defined as the sum of van der Waals surface area (VSA) of atoms whose property *X* lies in the range *Y*. In particular, PEOE_VSA13 and EState_VSA2 are related to the 3-dimensional distribution of electrons and are calculated using the partial equalization of orbital electronegativities (PEOE) method^24^ and electrotopological state index (EState) method^25^, respectively. Moreover, JGI2 is also a topological feature, which is computed by a 2-ordered mean topological charge. After optimizing the number of features (see Supplementary Information S3), the best predictive performances (MAE: 1.30, R^2^: 0.92) were obtained with the top 25 features, which are comparable to the predictive performance of the best *β*_1_ model. Figure 4b shows the parity plot between true and predicted multi-order *β*_*n*_ values of the validation samples using the best GPR model, indicating the high generalizability of our model again.

## Discussion

In this section, we discuss the important features for predicting *β*_1_ and multi-order *β*_*n*_. As a summary of the results obtained from the sensitivity analysis of the GPR model for predicting *β*_1_, electronegativity- or ionic-related features are sensitive to *β*_1_. In principle, when the electron polarization between the cation and the element at the coordination site of the ligand is small, a strong coordination bond is formed between them^2^. The electron distribution between them is then determined not only by the difference in electronegativities but also by the size of the cation. For *β*_1_, the Coulomb interaction between the cation and negatively charged ligand assists the formation of stable M-L complexes. Therefore, as shown in Fig. 2a, it is quite reasonable that features relevant to the electronegativity and ionic properties of both metals and ligands exhibited high relevance scores for predicting *β*_1_. In addition, we believe that these results were successfully obtained thanks to using experimental data for various cations. Given that the electronegativities of lanthanides are very similar, we recognize that their importance was underestimated in Chaube et al*.*^8^. However, because PEOE_VSA2, which was the most important feature in their study, is also related to electronegativity^24^, our results do not deviate from the findings of the previous studies.

Next, we focus on the relationship between multi-order *β*_*n*_ and *β*_1_. Because the *n*-th equilibrium constant *K*_*n*_ satisfies the relationship of $K_{1} > K_{2} > \cdots > K_{n}$, one can derive the following universal inequality:

4 $$ \begin{array}{*{20}c} {\beta_{n - 1} < \beta_{n} < n\beta_{1} .} \\ \end{array} $$

Equation 4 implies that the ratio *β*_*n*_/*β*_1_ is always larger than 1 and *β*_*n-1*_/*β*_1_ is smaller than *n*, which is observed in Fig. 3, with a few exceptions. Considering *β*_1_ reflects the cation–ligand binding strength to some degree, this suggests that a strong correlation between *β*_*n*_ and *β*_1_ is one of the intrinsic properties in the formation of complexes. In addition, the fact that VSA-related features are important for the multi-order *β*_*n*_ model is presumably because 3-dimensional structures such as steric hindrance are more influential than in the case of forming M-L complexes. Finally, we would like to mention the relationship between *β*_*i*_ and *β*_*j*_ (*i, j* > 1). As shown in Fig. 3, the multi-order stability constants have a linear dependence on *β*_1_, which might also mean the linear relationship between *β*_*i*_ and *β*_*j*_. We believe that these empirical trends can be useful to roughly predict stability constants for M-L_n_ complexes, which became soluble only when multiple ligands are coordinated.

## Conclusion

In this study, we developed two machine-learning models: one for predicting the first overall stability constant *β*_1_ and the other for predicting the multi-order overall stability constant *β*_*n*_. Using a very large training dataset, the developed models covered more than 50 cations, realizing the high generalizability of our models. Note that this is the first time a machine-learning model was created to predict the multi-order overall stability constant. Moreover, the relevance scores of features for both cations and ligands are quantitatively evaluated through sensitivity analysis to improve the interpretability of our models. Consequently, the most relevant features are consistent with physical understanding for complex formation. We believe that our findings are useful for the design and screening of new ligands for various applications. In particular, because it was concluded that the predicted *β*_1_ value was the most important property to predict multi-order *β*_*n*_ of the corresponding M-L pair, further development of the *β*_1_ model is expected to be necessary in the future. Finally, we would like to mention the advantages and disadvantages of our GPR models. One of the advantages is efficient searching for new ligands through Bayesian optimization, which is a topic we will study in the future. This is due to the fact that prediction uncertainty is quantified by GPR model. However, our models still cannot be applied to some cations, such as NH_4_^+^ and UO_2_^+^ because we focused on only single cations in this study. The descriptor for these cations may be prepared by averaging features of elements in them. By solving these remaining issues, we expect to realize a machine-learning model for predicting arbitrary complexes.

## Methods

### Dataset preparation

The experimental values of the *n*-th overall stability constants *β*_n_ for the M:L = 1:*n* complexes (*n* = 1–6) and experimental conditions were collected from the NIST Critically Selected Stability Constants of Metal Complexes Database^26^ and various literature^27–58^. In this study, data for several heavy metals (*i.e.*, Am, Cm, Cf, Bk, Es, Fm, and Md) or whose ligands contain elements such as Te, Se, As, Mn, Co, Fe, W, Mo, Cr, and Re were excluded due to the difficulties in making descriptors. Moreover, we collected experimental data according to the following priorities: data with temperature of 25 °C and ionic strength of 0.1 > data with temperature of 25 °C and any ionic strength > data with any temperature and ionic strength of 0.1 > data with the maximum overall stability constant. In the case of duplicates, the data with the largest overall stability constant was employed. Consequently, 19,810 M-L_n_ complexes remained, which consisted of 57 cations and 2706 ligands. The chemical structure of ligands is represented by SMILES (Simplified Molecular Input Line Entry System).

### Feature engineering

Following the previous study^8^, we used cation properties, ligand compositional and topological features, and experimental conditions, namely temperature and ionic strength, as the machine-learning descriptors for predicting *β*_1_. For cation descriptors, we initially selected 12 element-level features, such as cation charge, atomic number, melting point, molar specific heat capacity, ionic radius, polarizability, electron affinity, Pauling electronegativity, and numbers of unfilled electrons in s, p, d, and f orbitals^59,60^. We used molecular descriptor calculation software Mordred to generate compositional and topological descriptors for ligands^61^. In addition, we prepared the molecular charge of ligands in an aqueous solution as one of the ligand features. After removing features that have only a single value or null value, 587 ligand features remained. Subsequently, we calculated the Pearson correlation coefficient of the pair of ligand features *i* and *j*, *Corr(i, j)*, and if the absolute value of *Corr(i, j)* is greater than 0.7, we excluded the feature *j*. Furthermore, to avoid multicollinearity among features, we iteratively removed the feature with the largest variance inflation factor (VIF) score until the VIF score for all features became less than 4. In the case of predicting multi-order *β*_*n*_, we employed the predicted *β*_1_, the standard deviation of the predicted *β*_1_, and the charge of M-L complex, namely the sum of the cation charge and molecular charge, as the descriptor for M-L complex. The descriptor for ligands consisted of ligand features that were not used in the best *β*_1_ GPR model and the number of ligands to be additionally coordinated to the M-L complex, namely *n*−1. After feature engineering, the shapes of the final datasets for predicting *β*_1_ and multi-order *β*_*n*_ were 13,559 data × 118 features and 6251 data × 60 features, respectively.

### Gaussian process regression

In GPR, a similarity between data ***x***_*i*_ and ***x***_*j*_ is measured by the kernel, such as *k*(***x***_*i*_, ***x***_*j*_), which in turn defines a covariance matrix. Therefore, GPR is one of the powerful techniques because it naturally quantifies predicted values and their uncertainties. A well-known kernel choice is a Matérn kernel with *ν* = 3/2^62,63^, which is described as follows:

5 $$ \begin{array}{*{20}c} {k\left( {{\varvec{x}}_{i} , {\varvec{x}}_{j} } \right) = \sigma^{2} \left( {1 + \frac{\sqrt 3 r}{l}} \right)\exp \left( { - \frac{\sqrt 3 r}{l}} \right) ,} \\ \end{array} $$

where *σ*, *l*, and *r* are hyperparameters to represent the signal amplitude, length scale referring the relevance of features, and the Euclidean distance between data ***x***_*i*_ and ***x***_*j*_. As shown in Eq. (5), the usual Matérn kernel with *ν* = 3/2 has a single length scale parameter *l*. However, in this study, considering that the relevance of each descriptor should be different, the Matérn kernel with *ν* = 3/2 is modified with the automatic relevance determination (ARD) structure as follows:

6 $$ \begin{array}{*{20}c} {k\left( {{\varvec{x}}_{i} , {\varvec{x}}_{j} } \right) = \sigma^{2} \left( {1 + \sqrt 3 r_{{{\text{ARD}}}} } \right)\exp \left( { - \sqrt 3 r_{{{\text{ARD}}}} } \right) ,} \\ \end{array} $$

7 $$ \begin{array}{*{20}c} {r_{{{\text{ARD}}}} = \sqrt {\mathop \sum \limits_{m = 1}^{d} \frac{{\left( {x_{im} - x_{jm} } \right)^{2} }}{{l_{m}^{2} }}} ,} \\ \end{array} $$

where *d* is the dimension of a descriptor. Our GPR modeling was performed using PyTorch^64^ and GPytorch^65^.

## Supplementary Information

Supplementary Information.

## Data Availability

The full results of feature relevance calculated by sensitivity analysis and the details of feature optimization for the GPR model to predict multi-ligand stability constants are provided in Supplementary Information. Additional information regarding this study is available from the corresponding authors upon reasonable request.

## References

[1] Kanani, N. *Electroplating: Basic Principles, Processes and Practice* 1st edition (Elsevier, 2004).

[2] Singh, J., Srivastava, A. N., Singh, N. & Singh, A. *Stability Constants of Metal Complexes in Solution.* in *Stability and Applications of Coordination Compounds* (ed. Srivastava, A. N.) (IntechOpen, 2019).

[3] Treybal, R. E. *Mass transfer Operations* (Springer, 1980).

[4] Bruijnincx PCA, Sadler PJ (2008). New trends for metal complexes with anticancer activity. Curr. Opin. Chem. Biol..

[5] Dimmock PW, Warwick P, Robbins RA (1995). Approaches to predicting stability constants. Analyst.

[6] Janet JP, Kulik HJ (2017). Predicting electronic structure properties of transition metal complexes with neural networks. Chem. Sci..

[7] Meyer B, Sawatlon B, Heinen S, Anatole von Lilienfeld O, Corminboeuf C (2018). Machine learning meets volcano plots: computational discovery of cross-coupling catalysts. Chem. Sci..

[8] Chaube S, Goverapet Srinivasan S, Rai B (2020). Applied machine learning for predicting the lanthanide-ligand binding affinities. Sci. Rep..

[9] Solov’ev V, Kireeva N, Ovchinnikova S, Tsivadze A (2015). The complexation of metal ions with various organic ligands in water prediction of stability constants by QSPR ensemble modelling. J. Incl. Phenom. Macrocycl. Chem..

[10] Tetko IV, Solovev VP, Antonov AV (2006). Benchmarking of linear and nonlinear approaches for quantitative structure-property relationship studies of metal complexation with ionophores. J. Chem. Inf. Model..

[11] Solov’ev V, Marcou G, Tsivadze A, Varnek A (2012). Complexation of Mn^2+^, Fe^2+^, Y^3+^, La^3+^, Pb^2+^, and UO_2_^2+^ with organic ligands: QSPR ensemble modeling of stability constants. Ind. Eng. Chem. Res..

[12] Solov’ev VP, Tsivadze AY, Varnek AA (2012). New approach for accurate QSPR modeling of metal complexation: Application to stability constants of complexes of lanthanide ions Ln^3+^ Ag^+^, Zn^2+^, Cd^2+^ and Hg^2+^ with organic ligands in water. Macroheterocycles.

[13] Solv’ev VP, Kireeva N, Tsivadze Y, Varnek A (2013). QSPR ensemble modelling of alkaline-earth metal complexation. J. Incl. Phenom. Macrocycl. Chem..

[14] Solv’ev V (2012). Stability constants of complexes of Zn^2+^, Cd^2+^, and Hg^2+^ with organic ligands: QSPR consensus modeling and design of new metal binders. J. Incl. Phenom. Macrocycl. Chem..

[15] Baskin II, Solov’ev VP, Bagatur’yants AA, Varnek A (2017). Predictive cartography of metal binders using generative topographic mapping. J. Comput. Aided. Mol. Des..

[16] Quang NM, Nhung NTA, Tat PV (2019). An insight QSPR-based prediction model for stability constants of metal-thiosemicarbazone complexes using MLR and ANN methods. Vietnam J. Chem..

[17] Shiri F, Salahinejad M, Momeni-Mooguei N, Sanchooli M (2021). Predicting stability constants of transition metals; Y^3+^, La^3+^, and UO_2_^2+^ with organic ligands using the 3D-QSPR methodology. J. Recept. Signal Transduct. Res..

[18] Solov’ev V, Varnek A, Tsivadze A (2014). QSPR ensemble modelling of the 1:1 and 1:2 complexation of Co^2+^, Ni^2+^, and Cu^2+^ with organic ligands: relationships between stability constants. J. Comput. Aided. Mol. Des..

[19] Deringer VL, Bartók AP, Bernstein N, Wilkins DM, Ceriotti M, Csányi G (2021). Gaussian process regression for materials and molecules. Chem. Rev..

[20] Motoyama Y, Tamura R, Yoshimi K, Terayama K, Ueno Y, Tsuda K (2022). Bayesian optimization package: PHYSBO. Comput. Phys. Commun..

[21] Zhang H (2004). Inconsistent estimation and asymptotically equal interpolations in model-based geostatistics. J. Am. Stat. Assoc..

[22] Piironen, J. & Vehtari, A. Projection predictive model selection for Gaussian processes. *2016 IEEE 26th International Workshop on Machine Learning for Signal Processing (MLSP)*, **2016**, 1–6 (2016).

[23] Paananen T, Piironen J, Andersen MR, Vehtari A (2019). Variable selection for Gaussian processes via sensitivity analysis of the posterior predictive distribution. Proc. 22nd Int Conf. Artig. Intell. Statist..

[24] Gasteiger J, Marsili M (1980). Iterative partial equalization of orbital electronegativity-A rapid access to atomic charges. Tetrahedron.

[25] Hall LH, Kier LB (1995). Electrotopological state indices for atom types: A novel combination of electronic, topological, and valence state information. J. Chem. Inf. Comput. Sci..

[26] Smith, R. M. & Martell, A. E. *NIST Critically Selected Stability Constants of Metal Complexes Database (NIST Standard Reference Database 46)*. version 8.0, (National Institute of Science and Technology, Gaithersburg, MD, 2004). https://www.nist.gov/srd/nist46. Accessed 1 March 2022.

[27] Fernandez-Botello A, Griesser R, Holý A, Moreno V, Sigel H (2005). Acid−base and metal-ion-binding properties of 9-[2-(2-Phosphonoethoxy)ethyl]adenine (PEEA), a relative of the antiviral nucleotide analogue 9-[2-(Phosphonomethoxy)ethyl]adenine (PMEA). An exercise on the quantification of isomeric complex equilibria in solution. Inorg. Chem..

[28] Kapinos LE, Holý A, Günter J, Sigel H (2001). Metal ion-binding properties of 1-Methyl-4-aminobenzimidazole (=9-Methyl-1,3-dideazaadenine) and 1,4-Dimethylbenzimidazole (=6,9-Dimethyl-1,3-dideazapurine). Quantification of the steric effect of the 6-Amino group on metal ion binding at the N7 site of the adenine residue. Inorg. Chem..

[29] Melton DL, VanDerveer DG, Hancock RD (2006). Complexes of greatly enhanced thermodynamic stability and metal ion size-based selectivity, formed by the highly preorganized non-macrocyclic ligand 1,10-Phenanthroline-2,9-dicarboxylic Acid. A thermodynamic and crystallographic study. Inorg. Chem..

[30] Sigel H, Da Costa CP, Song B, Carloni P, Gregáň F (1999). Stability and structure of metal ion complexes formed in solution with acetyl phosphate and acetonylphosphonate: Quantification of isomeric equilibria. J. Am. Chem. Soc..

[31] Kálmán FK (2008). Synthesis, Potentiometric, Kinetic, and NMR Studies of 1,4,7,10-Tetraazacyclododecane-1,7-bis(acetic acid)-4,10-bis(methylenephosphonic acid) (DO2A2P) and its Complexes with Ca(II), Cu(II), Zn(II) and Lanthanide(III) Ions. Inorg. Chem..

[32] Nonat A, Gateau C, Fries PH, Mazzanti M (2006). Lanthanide complexes of a picolinate ligand derived from 1,4,7-Triazacyclononane with potential application in magnetic resonance imaging and time-resolved luminescence imaging. Chem. Eur. J..

[33] Kotek J (2006). Study of thermodynamic and kinetic stability of transition metal and lanthanide complexes of DTPA analogues with a phosphorus acid pendant arm. Eur. J. Inorg. Chem..

[34] Rodríguez L (2008). Anion detection by fluorescent Zn(II) complexes of functionalized polyamine ligands. Inorg. Chem..

[35] Aragoni MC (2005). Coordination chemistry of N-aminopropyl pendant arm derivatives of mixed N/S-, and N/S/O-donor macrocycles, and construction of selective fluorimetric chemosensors for heavy metal ions. Dalton Trans..

[36] Caltagirone C (2003). Redox chemosensors: coordination chemistry towards Cu^II^, Zn^II^, Cd^II^, Hg^II^, and Pb^II^ of 1-aza-4,10-dithia-7-oxacyclododecane ([12]aneNS2O) and its N-ferrocenylmethyl derivative. Dalton Trans..

[37] Bazzicalupi C (2004). Protonation and coordination properties towards Zn(II), Cd(II) and Hg(II) of a phenanthroline-containing macrocycle with an ethylamino pendant arm. Dalton Trans..

[38] Blake AJ (2004). A new pyridine-based 12-membered macrocycle functionalised with different fluorescent subunits; coordination chemistry towards Cu^II^, Zn^II^, Cd^II^, Hg^II^, and Pb^II^. Dalton Trans..

[39] Baranyai Z, Bombieri G, Meneghetti F, Tei L, Botta M (2009). A solution thermodynamic study of the Cu(II) and Zn(II) complexes of EBTA: X-ray crystal structure of the dimeric complex [Cu_2_(EBTA)(H_2_O)_3_]_2_. Inorg. Chim. Acta.

[40] Miguirditchian M (2005). Thermodynamic Study of the Complexation of Trivalent Actinide and Lanthanide Cations by ADPTZ, a Tridentate N-Donor Ligand. Inorg. Chem..

[41] Kobayashi T (2010). Effect of the introduction of amide oxygen into 1,10-Phenanthroline on the extraction and complexation of trivalent lanthanide in acidic condition. Sep. Sci. Technol..

[42] Miguirditchian M (2006). Complexation of Lanthanide(III) and Actinide(III) cations with tridentate nitrogen-donor ligands: A luminescence and spectrophotometric study. Nucl. Sci. Eng..

[43] Ogden MD, Sinkov SI, Meier GP, Lumetta GJ, Nash KL (2012). Complexation of N_4_-Tetradentate ligands with Nd(III) and Am(III). J. Solut. Chem..

[44] Merrill D, Hancock RD (2011). Metal ion selectivities of the highly preorganized tetradentate ligand 1,10-phenanthroline-2,9-dicarboxamide with lanthanide(III) ions and some actinide ions. Radiochim. Acta.

[45] Reddy KH, Prasad NBL, Reddy TS (2003). Analytical properties of 1-phenyl-1,2-propanedione-2-oxime thiosemicarbazone: simultaneous spectrophotometric determination of copper(II) and nickel(II) in edible oils and seeds. Talanta.

[46] Veeranna V, Rao VS, Laxmi VV, Varalankshmi TR (2013). Simultaneous second order derivative spectrophotometric determination of cadmium and cobalt using furfuraldehyde Thiosemicarbazone (FFTSC). Res. J. Phyarm. Tech..

[47] Atalay T, Özkan E (1994). Evaluation of thermodynamic parameters and stability constants of Cu(II), Ag(I) and Hg(II) complexes of 2-methylindole-3-carboxaldehyde thiosemicarbazone. Thermochim. Acta.

[48] Sharma SRK, Sindhwani SK (1992). Thermal studies on the chelation behavior of biologically active 2-hydroxy-1-naphthaldehyde thiosemicarbazone (HNATS) towards bivalent metal ions: A potentiometric study. Thermochim. Acta.

[49] Drahoš B (2011). Mn^2+^ complexes with 12-membered pyridine based macrocycles bearing carboxylate or phosphonate pendant arm: Crystallographic, thermodynamic, kinetic, redox, and ^1^H/^17^O relaxation studies. Inorg. Chem..

[50] Drahoš B, Kotek J, Hermann P, Lukeš I, Toth É (2010). Mn^2+^ Complexes with pyridine-containing 15-membered macrocycles: thermodynamic, kinetic, crystallographic, and ^1^H/^17^O relaxation studies. Inorg. Chem..

[51] Svobodová I (2006). Thermodynamic, kinetic and solid-state study of divalent metal complexes of 1,4,8,11-tetraazacyclotetradecane (cyclam) bearing two trans (1,8-)methylphosphonic acid pendant arms. Dalton Trans..

[52] Bazzicalupi C (2006). Basicity and coordination properties of a new phenanthroline-based bis-macrocyclic receptor. Dalton Trans..

[53] Yamada H, Hayashi H, Yasui T (2006). Utility of 1-Octanol/Octane mixed solvents for the solvent extraction of Aluminum(III), Gallium(III), and Indium(III) with 8-Quinolinol. Anal. Sci..

[54] Jurchen KMC, Raymond KN (2006). A bidentate terephthalamide ligand, TAMmeg, as an entry into terephthalamide-containing therapeutic iron chelating agents. Inorg. Chem..

[55] Dertz EA, Xu J, Raymond KN (2006). Tren-based analogs of bacillibactin: structure and stability. Inorg. Chem..

[56] Gephart Iii RT, Williams NJ, Reibenspies JH, De Sousa AS, Hancock RD (2008). Metal ion complexing properties of the highly preorganized ligand 2, 9-bis (hydroxymethyl)-1, 10-phenanthroline: A crystallographic and thermodynamic study. Inorg. Chem..

[57] Hancock RD, De Sousa AS, Walton GB, Reibenspies JH (2007). Metal-ion selectivity produced by C-Alkyl substituents on the bridges of chelating ligands: The importance of short H−H nonbonded van der waals contacts in controlling metal-ion selectivity. A thermodynamic, molecular mechanics, and crystallographic study. Inorg. Chem..

[58] Nagy NV (2012). Copper(II)-binding ability of stereoisomeric cis- and trans-2-Aminocyclohexanecarboxylic Acid–L-Phenylalanine Dipeptides. A combined CW/Pulsed EPR and DFT study. Inorg. Chem..

[59] Yamada H (2019). Predicting materials properties with little data using shotgun transfer learning. ACS Cent. Sci..

[60] Shannon RD (1976). Revised effective ionic radii and systematic studies of interatomic distances in halides and chalcogenides. Acta Crist. A.

[61] Moriwaki H, Tian Y-S, Kawashita N, Takagi T (2018). Mordred: a molecular descriptor calculator. J. Cheminformatics.

[62] Williams, C. K. & Rasmussen, C. E. *Gaussian Processes for Machine Learning* Vol. 2 (MIT Press, 2006).

[63] Noack MM (2020). Autonomous materials discovery driven by Gaussian process regression with inhomogeneous measurement noise and anisotropic kernels. Sci. Rep..

[64] Paszke A (2019). PyTorch: An imperative style, high-performance deep learning library. Adv. Neural Inf. Process. Syst..

[65] Gardner JR, Pleiss G, Weinberger KQ, Bindel D, Wilson AG (2018). GPyTorch: Blackbox matrix-matrix Gaussian process inference with GPU acceleration. Adv. Neural Inf. Process. Syst..

