# Supplementary material for: Machine learning-based analysis of overall stability constants of metal–ligand complexes
Source: Sci Rep. 2022 Jul 25;12:11159. doi: 10.1038/s41598-022-15300-9 (PMC9314427; doi:10.1038/s41598-022-15300-9)
Supplement: Supplementary file 1 — Supplementary Information. [file 41598_2022_15300_MOESM1_ESM.docx]

**Supplementary Information**

**Machine learning-based analysis of overall stability constants of metal-ligand complexes**

Kaito Kanahashi, Makoto Urushihara, and Kenji Yamaguchi

Innovation Center, Mitsubishi Materials Corporation, 1002-14 Mukohyama, Naka, Ibaraki 311-0102, Japan

**Contents**

1. **Distribution of molecular weights**
2. **Full results of feature relevance for predicting *β*_1_**
3. **Feature optimization of our models**
4. **Full results of feature relevance for predicting multi-order *β_n_***
5. **Cross-validation results of the feature optimized models**

**S1. Distribution of molecular weights**

Figures S1a and S1b show the distributions of the molecular weights of the ligands for *β*_1_ (2654 ligands) and multi-order *β_n_* (*n*=2-6, 1210 ligands), respectively. The average molecular weights for them are 232 and 168, respectively. As shown in Fig. S1, the chemical space of our training data is composed of a wide variety of ligands.


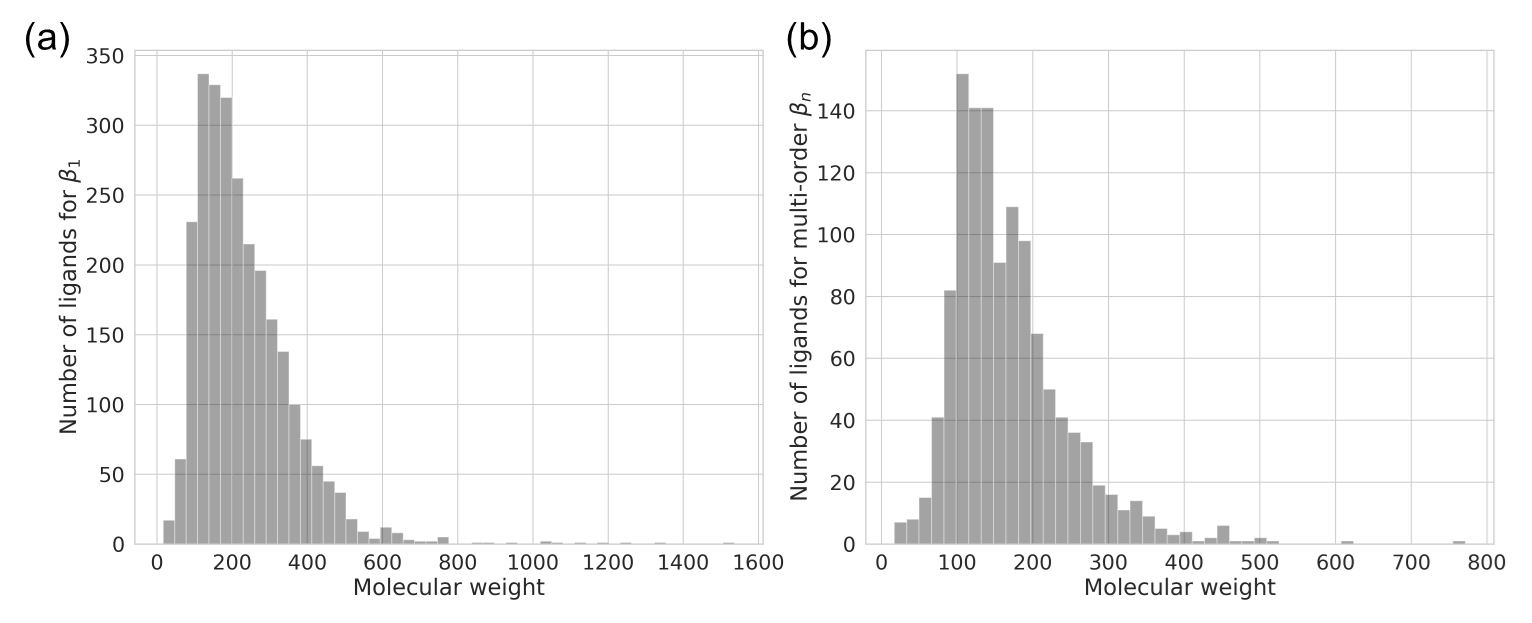


**Figure S1.** The distributions of molecular weights of ligands for (a) *β*_1_ (2654 ligands) and (b) multi-order *β_n_* (*n*=2-6, 1210 ligands).

**S2. Full results of feature relevance for predicting *β*_1_**

Table S1 shows the full results of standardized feature relevance for predicting *β*_1_. The relevance score was computed by sensitivity analysis using the Kullback–Leibler divergence as a measure [1]. The standardized inverse of ARD length-scale is also listed in Table S1. Refer to previous works for the definitions of each feature [2-4]

**Table S1.** Full results of feature relevance for predicting *β*_1_. Relevance scores calculated by sensitivity analysis and the inverse of ARD length-scale are referred to as Rel_KL and Rel_ARD, respectively.

| No. | feature | Standardized Rel_KL | Standardized Rel_ARD |
| --- | --- | --- | --- |
| 1 | en_pauling | 1.709.E-01 | 1.364.E-02 |
| 2 | AATS0Z | 1.510.E-01 | 2.242.E-02 |
| 3 | molecular_charge | 1.434.E-01 | 8.921.E-03 |
| 4 | cation_charge | 1.300.E-01 | 1.028.E-02 |
| 5 | AATS0i | 3.941.E-02 | 7.208.E-03 |
| 6 | ionic_radius | 3.145.E-02 | 5.049.E-03 |
| 7 | NssO | 2.587.E-02 | 4.861.E-03 |
| 8 | ATSC3se | 2.382.E-02 | 4.129.E-03 |
| 9 | NssNH | 2.113.E-02 | 5.121.E-03 |
| 10 | melting_point | 1.759.E-02 | 3.517.E-03 |
| 11 | IC0 | 1.533.E-02 | 7.365.E-03 |
| 12 | atomic_number | 1.529.E-02 | 4.876.E-03 |
| 13 | ATSC3m | 1.443.E-02 | 3.015.E-03 |
| 14 | NsNH2 | 1.250.E-02 | 3.240.E-03 |
| 15 | JGI1 | 1.131.E-02 | 3.738.E-03 |
| 16 | PEOE_VSA12 | 9.360.E-03 | 2.827.E-03 |
| 17 | num_s_unfilled | 7.755.E-03 | 3.465.E-03 |
| 18 | ATSC7p | 5.575.E-03 | 2.887.E-03 |
| 19 | ATSC2Z | 4.919.E-03 | 3.267.E-03 |
| 20 | num_d_unfilled | 4.695.E-03 | 3.530.E-03 |
| 21 | SMR_VSA9 | 4.270.E-03 | 2.175.E-03 |
| 22 | ion_strength | 4.121.E-03 | 3.101.E-03 |
| 23 | electron_affinity | 4.075.E-03 | 4.057.E-03 |
| 24 | n7HRing | 4.033.E-03 | 2.646.E-02 |
| 25 | n11Ring | 4.023.E-03 | 2.553.E-02 |
| 26 | n9Ring | 3.941.E-03 | 2.567.E-02 |
| 27 | n12Ring | 3.912.E-03 | 2.433.E-03 |
| 28 | NaaN | 3.828.E-03 | 2.477.E-03 |
| 29 | n4ARing | 3.799.E-03 | 2.857.E-02 |
| 30 | n11FRing | 3.708.E-03 | 2.762.E-02 |
| 31 | NaaS | 3.606.E-03 | 3.123.E-02 |
| 32 | n8Ring | 3.464.E-03 | 2.437.E-02 |
| 33 | SdssS | 3.417.E-03 | 2.435.E-02 |
| 34 | NssPH | 3.291.E-03 | 2.103.E-02 |
| 35 | n8FRing | 3.289.E-03 | 2.114.E-02 |
| 36 | C1SP1 | 3.284.E-03 | 2.386.E-02 |
| 37 | NdNH | 3.236.E-03 | 5.008.E-02 |
| 38 | n10FAHRing | 3.222.E-03 | 2.077.E-02 |
| 39 | n10Ring | 3.219.E-03 | 2.083.E-02 |
| 40 | C2SP1 | 3.189.E-03 | 2.352.E-02 |
| 41 | nG12FaRing | 3.174.E-03 | 2.160.E-02 |
| 42 | NtCH | 3.155.E-03 | 2.355.E-02 |
| 43 | nBondsT | 3.134.E-03 | 2.327.E-02 |
| 44 | n10FARing | 3.015.E-03 | 2.277.E-02 |
| 45 | n4HRing | 2.982.E-03 | 2.039.E-02 |
| 46 | PEOE_VSA7 | 2.980.E-03 | 2.990.E-03 |
| 47 | NssssN | 2.933.E-03 | 2.196.E-02 |
| 48 | SsssB | 2.905.E-03 | 3.661.E-02 |
| 49 | NsCH3 | 2.855.E-03 | 2.831.E-03 |
| 50 | n12FRing | 2.790.E-03 | 4.089.E-02 |
| 51 | n4aRing | 2.356.E-03 | 1.608.E-02 |
| 52 | n9FARing | 2.355.E-03 | 1.898.E-02 |
| 53 | ATSC7are | 2.324.E-03 | 3.104.E-03 |
| 54 | n7Ring | 2.250.E-03 | 1.549.E-02 |
| 55 | NssNH2 | 2.229.E-03 | 1.868.E-02 |
| 56 | NsssNH | 2.066.E-03 | 1.669.E-02 |
| 57 | SssssSi | 2.051.E-03 | 1.752.E-02 |
| 58 | temperature | 1.854.E-03 | 3.746.E-03 |
| 59 | ATSC8m | 1.804.E-03 | 2.970.E-03 |
| 60 | SlogP_VSA10 | 1.443.E-03 | 2.697.E-03 |
| 61 | JGI8 | 1.424.E-03 | 3.267.E-03 |
| 62 | EState_VSA5 | 1.332.E-03 | 2.632.E-03 |
| 63 | EState_VSA6 | 1.274.E-03 | 2.754.E-03 |
| 64 | JGI10 | 1.216.E-03 | 3.186.E-03 |
| 65 | EState_VSA2 | 1.102.E-03 | 3.144.E-03 |
| 66 | JGI4 | 9.765.E-04 | 3.450.E-03 |
| 67 | heat_capacity_molar | 9.581.E-04 | 3.511.E-03 |
| 68 | PEOE_VSA10 | 9.035.E-04 | 3.128.E-03 |
| 69 | VSA_EState7 | 8.991.E-04 | 2.840.E-03 |
| 70 | ATSC8i | 8.471.E-04 | 2.719.E-03 |
| 71 | JGI9 | 7.827.E-04 | 3.313.E-03 |
| 72 | JGI2 | 7.719.E-04 | 3.509.E-03 |
| 73 | SlogP_VSA4 | 7.626.E-04 | 1.908.E-03 |
| 74 | ATSC7m | 7.122.E-04 | 2.864.E-03 |
| 75 | EState_VSA3 | 5.684.E-04 | 3.076.E-03 |
| 76 | JGI5 | 5.636.E-04 | 3.287.E-03 |
| 77 | PEOE_VSA6 | 5.138.E-04 | 2.874.E-03 |
| 78 | num_p_unfilled | 4.983.E-04 | 2.439.E-03 |
| 79 | num_f_unfilled | 4.626.E-04 | 3.812.E-03 |
| 80 | JGI3 | 4.395.E-04 | 2.859.E-03 |
| 81 | NsSH | 3.763.E-04 | 1.549.E-03 |
| 82 | ATSC3p | 3.432.E-04 | 2.384.E-03 |
| 83 | SsssP | 2.880.E-04 | 2.149.E-03 |
| 84 | EState_VSA7 | 2.530.E-04 | 2.348.E-03 |
| 85 | JGI7 | 2.009.E-04 | 3.034.E-03 |
| 86 | nBridgehead | 1.942.E-04 | 1.835.E-03 |
| 87 | nI | 1.316.E-04 | 1.529.E-03 |
| 88 | n5ARing | 1.244.E-04 | 2.757.E-03 |
| 89 | NdsN | 1.190.E-04 | 2.622.E-03 |
| 90 | PEOE_VSA4 | 1.106.E-04 | 2.342.E-03 |
| 91 | SlogP_VSA7 | 1.077.E-04 | 1.096.E-03 |
| 92 | PEOE_VSA3 | 1.076.E-04 | 2.695.E-03 |
| 93 | nBr | 1.070.E-04 | 1.174.E-03 |
| 94 | C3SP2 | 1.046.E-04 | 1.912.E-03 |
| 95 | SaasC | 9.827.E-05 | 1.816.E-03 |
| 96 | n6ARing | 7.411.E-05 | 2.630.E-03 |
| 97 | SddssS | 6.561.E-05 | 1.414.E-03 |
| 98 | nCl | 6.155.E-05 | 1.153.E-03 |
| 99 | C3SP3 | 5.955.E-05 | 2.323.E-03 |
| 100 | SsssCH | 5.618.E-05 | 2.587.E-03 |
| 101 | ECIndex | 5.608.E-05 | 6.393.E-04 |
| 102 | NaasN | 5.545.E-05 | 1.707.E-03 |
| 103 | NdCH2 | 4.639.E-05 | 1.399.E-03 |
| 104 | PEOE_VSA5 | 4.303.E-05 | 1.640.E-03 |
| 105 | Xch-3d | 4.021.E-05 | 8.230.E-04 |
| 106 | NdS | 3.816.E-05 | 2.958.E-03 |
| 107 | n6AHRing | 3.746.E-05 | 1.993.E-03 |
| 108 | NaaNH | 3.649.E-05 | 1.949.E-03 |
| 109 | n9FRing | 3.466.E-05 | 2.022.E-03 |
| 110 | C4SP3 | 3.222.E-05 | 1.411.E-03 |
| 111 | NdsCH | 3.185.E-05 | 1.623.E-03 |
| 112 | SddsN | 3.105.E-05 | 3.459.E-03 |
| 113 | NaaO | 2.975.E-05 | 1.520.E-03 |
| 114 | n10FHRing | 2.968.E-05 | 1.756.E-03 |
| 115 | PEOE_VSA11 | 2.802.E-05 | 1.727.E-03 |
| 116 | PEOE_VSA13 | 2.535.E-05 | 1.691.E-03 |
| 117 | SssssC | 1.784.E-05 | 2.078.E-03 |
| 118 | SssssN | 1.566.E-05 | 2.239.E-03 |

**S3. Feature optimization of our models**

In this study, we decided that appropriate validation samples need to satisfy two conditions: one is that their ligands are not included in training data and the other is that they are located near the edge of the applicability domain (AD) of our model. By doing this, we assume that it is possible to evaluate the generalizability of our model for data outside AD. Thus, we first visualized AD by the dimension reduction technique. Figure S2a shows the uniform manifold approximation and projection (UMAP) scatter plot of the dataset for predicting *β*_1_. UMAP is a non-linear dimension reduction technique that is suitable for dealing with large datasets [5]. As shown in Fig. S2a, the M-L complexes with similar ligands are located close together. Thus, Fig. S2a is one of the visualizations of the AD of our GPR model for predicting *β*_1_. Based on Fig. S2a, we calculated the Mahalanobis distance from the center of the AD for each data point and selected 20 M-L complexes that satisfy the above two conditions.

The feature optimization procedure for the *β_n_* GPR model is as follows. First, we visualize the AD using the dataset for predicting *β_n_*, as shown in Fig. S2b. Second, for each *β_n_*, we extracted the M-L_n_ complex with the longest Mahalanobis distance from the center of the applicability domain of the *β_n_* model. Finally, all data on the selected 5 ligands was extracted, and then the dataset for multi-order *β_n_* was separated into 6149 training and 102 validation data points. We thus successfully reproduced the situation for predicting *β_n_* of unknown ligands. Figure S2c shows the predictive performance for the validation data using the *β_n_* GPR model as a function of the descriptor dimension. The features were arranged in descending order of the relevance scores displayed in Fig. 4a in the manuscript. As shown in Fig. S2c, the best predictive performance was obtained with the top 25 features.


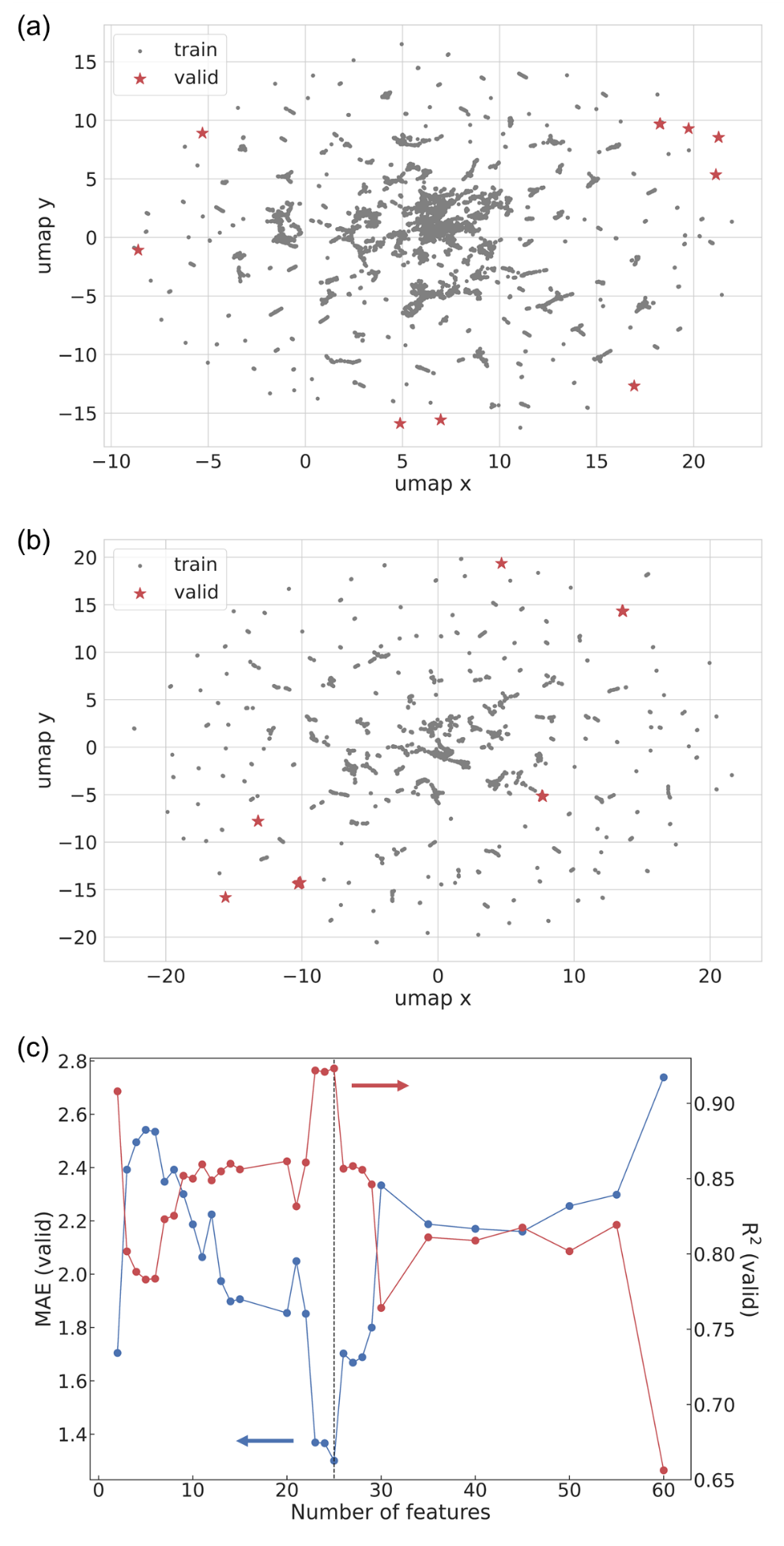


**Figure S2.** (a) Visualization of the dataset for *β*_1_ using UMAP. Data represented by gray circles and red stars were used for the training and valid data for feature optimization, respectively. (b) Visualization of the dataset for multi-order *β_n_* using UMAP. Data represented by gray circles and red stars were used for the training and valid data for feature optimization, respectively. (c) Predictive performance for the valid data as a function of the number of features. Features are arranged in descending order of relevance. The black dashed line corresponds to the top 25 features.

**S4. Full results of feature relevance for predicting *β_n_***

Table S2 shows the full results of standardized feature relevance for predicting *β_n_*. The relevance score was computed by sensitivity analysis using the Kullback–Leibler divergence as a measure [1]. The standardized inverse of ARD length-scale is also listed in Table S2.

**Table S2.** Full results of feature relevance for predicting *β_n_*. Relevance scores calculated by sensitivity analysis and the inverse of ARD length-scale are referred as Rel_KL and Rel_ARD, respectively.

| No. | feature | Standardized Rel_KL | Standardized Rel_ARD |
| --- | --- | --- | --- |
| 1 | predicted beta1 | 7.110.E-01 | 3.437.E-02 |
| 2 | n-1 | 1.677.E-01 | 1.543.E-02 |
| 3 | std. beta1 | 5.002.E-02 | 2.336.E-02 |
| 4 | charge of M-L complex | 2.261.E-02 | 1.620.E-02 |
| 5 | NaaO | 1.473.E-02 | 4.773.E-01 |
| 6 | nBridgehead | 7.150.E-03 | 5.646.E-02 |
| 7 | SlogP_VSA4 | 4.881.E-03 | 9.647.E-03 |
| 8 | JGI2 | 3.785.E-03 | 1.187.E-02 |
| 9 | PEOE_VSA13 | 2.898.E-03 | 8.851.E-03 |
| 10 | EState_VSA2 | 1.298.E-03 | 1.010.E-02 |
| 11 | NdsN | 1.266.E-03 | 6.749.E-03 |
| 12 | JGI5 | 9.610.E-04 | 1.126.E-02 |
| 13 | PEOE_VSA3 | 8.801.E-04 | 1.039.E-02 |
| 14 | JGI3 | 8.051.E-04 | 1.092.E-02 |
| 15 | NdS | 7.007.E-04 | 5.231.E-03 |
| 16 | PEOE_VSA6 | 6.526.E-04 | 7.458.E-03 |
| 17 | ATSC3p | 6.191.E-04 | 9.413.E-03 |
| 18 | SlogP_VSA10 | 5.559.E-04 | 8.953.E-03 |
| 19 | PEOE_VSA10 | 5.351.E-04 | 8.865.E-03 |
| 20 | EState_VSA5 | 4.256.E-04 | 9.323.E-03 |
| 21 | SddsN | 3.854.E-04 | 7.609.E-03 |
| 22 | SsssP | 3.816.E-04 | 3.212.E-03 |
| 23 | nI | 3.512.E-04 | 4.798.E-03 |
| 24 | VSA_EState7 | 3.423.E-04 | 9.151.E-03 |
| 25 | NsSH | 3.221.E-04 | 5.476.E-03 |
| 26 | SlogP_VSA7 | 3.063.E-04 | 2.027.E-03 |
| 27 | SssssN | 2.971.E-04 | 2.227.E-03 |
| 28 | nCl | 2.096.E-04 | 3.117.E-03 |
| 29 | JGI4 | 1.899.E-04 | 1.026.E-02 |
| 30 | nBr | 1.876.E-04 | 4.476.E-03 |
| 31 | JGI7 | 1.871.E-04 | 8.340.E-03 |
| 32 | JGI10 | 1.809.E-04 | 4.539.E-03 |
| 33 | SddssS | 1.760.E-04 | 4.847.E-03 |
| 34 | C4SP3 | 1.691.E-04 | 7.927.E-03 |
| 35 | PEOE_VSA11 | 1.631.E-04 | 7.929.E-03 |
| 36 | n6AHRing | 1.618.E-04 | 4.721.E-03 |
| 37 | ECIndex | 1.538.E-04 | 6.690.E-03 |
| 38 | n9FRing | 1.524.E-04 | 4.496.E-03 |
| 39 | JGI9 | 1.418.E-04 | 5.045.E-03 |
| 40 | ATSC7m | 1.359.E-04 | 8.539.E-03 |
| 41 | NdCH2 | 1.286.E-04 | 4.551.E-03 |
| 42 | EState_VSA3 | 1.195.E-04 | 8.752.E-03 |
| 43 | NaasN | 1.190.E-04 | 5.705.E-03 |
| 44 | NdsCH | 1.190.E-04 | 4.719.E-03 |
| 45 | JGI8 | 1.188.E-04 | 6.710.E-03 |
| 46 | PEOE_VSA5 | 1.180.E-04 | 7.191.E-03 |
| 47 | NaaNH | 1.125.E-04 | 8.087.E-03 |
| 48 | PEOE_VSA4 | 1.085.E-04 | 6.809.E-03 |
| 49 | SssssC | 9.987.E-05 | 7.766.E-03 |
| 50 | SaasC | 9.980.E-05 | 6.811.E-03 |
| 51 | EState_VSA6 | 9.922.E-05 | 8.498.E-03 |
| 52 | n5ARing | 9.242.E-05 | 6.984.E-03 |
| 53 | n6ARing | 9.047.E-05 | 5.330.E-03 |
| 54 | n10FHRing | 9.047.E-05 | 4.556.E-03 |
| 55 | C3SP2 | 8.751.E-05 | 6.619.E-03 |
| 56 | Xch-3d | 8.664.E-05 | 2.221.E-03 |
| 57 | EState_VSA7 | 8.585.E-05 | 7.363.E-03 |
| 58 | C3SP3 | 7.138.E-05 | 8.772.E-03 |
| 59 | SsssCH | 6.384.E-05 | 8.202.E-03 |
| 60 | ATSC8i | 5.707.E-05 | 6.731.E-03 |

**S5. Cross-validation results of the feature optimized models**

We also performed n-fold (n=4-10) cross-validations for feature-optimized GPR models to check their predictive performances. Figures S3a and S3b show the training dataset size dependence of prediction errors (R^2^: coefficient of determination, MAE: mean absolute error) of the feature-optimized GPR models for predicting *β*_1_ and multi-order *β_n_*, respectively. As shown in Fig. S3, both GPR models indicated good predictive performances, which further support the high generalizability of our GPR models.


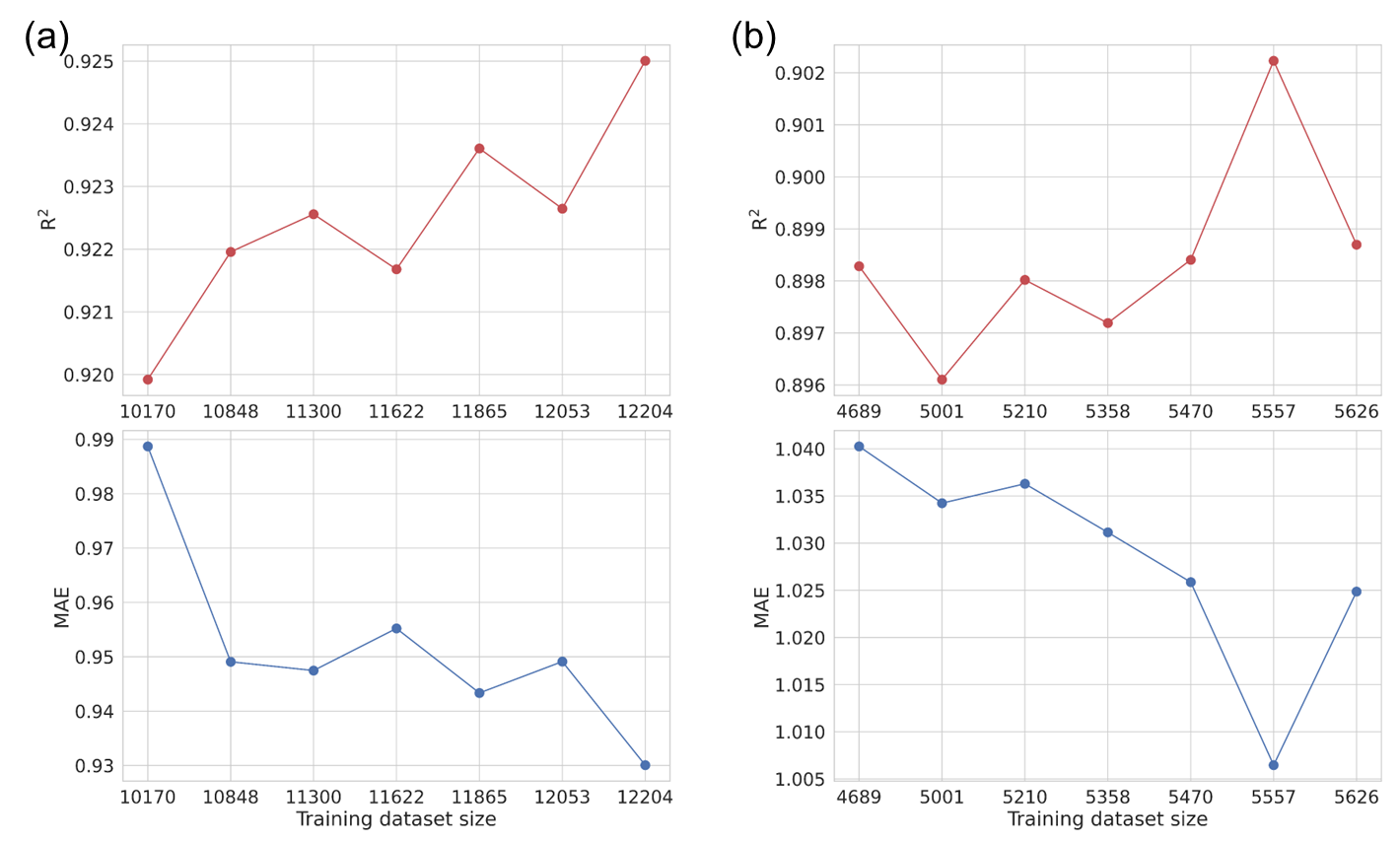


**Figure S3**. Summary of n-fold (n=4-10) cross-validation results. As the number of folds increases, the training dataset size increases. (a) The training dataset size dependence of prediction errors (R^2^: coefficient of determination, MAE: mean absolute error) of the GPR model for predicting *β*_1_. (b) The training dataset size dependence of prediction errors of the GPR model for predicting multi-order *β_n_*.

**Reference**

1. Paananen, T., Piironen, J., Andersen, M. R. & Vehtari, A. Variable selection for Gaussian processes via sensitivity analysis of the posterior predictive distribution. *Proc. 22^nd^ Int. Conf. Artig. Intell. Statist.* **89**, 1743-1752 (2019).
2. Yamada. H. *et al*. Predicting Materials Properties with Little Data Using Shotgun Transfer Learning. *ACS Cent. Sci*. **5**, 1717-1730 (2019).
3. Shannon, R. D. Revised effective ionic radii and systematic studies of interatomic distances in halides and chalcogenides. *Acta Crist. A* **32**, 751-767 (1976).
4. Moriwaki, H., Tian, Y.-S., Kawashita, N. & Takagi, T. Mordred: a molecular descriptor calculator. *J. Cheminformatics* **10**, 1-14 (2018).
5. McInnes, L., Healy, J. & Melville, J. UMAP: Uniform Manifold Approximation and Projection for Dimension Reduction. Preprint at <https://arxiv.org/abs1802.03426>.
